# Moderate hypoxia mitigates the physiological effects of high temperature on the tropical blue crab *Callinectes sapidus*

**DOI:** 10.3389/fphys.2022.1089164

**Published:** 2023-01-05

**Authors:** Adriana L. Garcia-Rueda, Maite Mascaro, Gabriela Rodriguez-Fuentes, Claudia P. Caamal-Monsreal, Fernando Diaz, Kurt Paschke, Carlos Rosas

**Affiliations:** ^1^ Posgrado en Ciencias del Mar y Limnologia, Universidad Nacional Autonoma de Mexico, Mexico City, Mexico; ^2^ Unidad Multidisciplinaria de Docencia e Investigacion Sisal (UMDI-Sisal), Facultad de Ciencias, Universidad Nacional Autonoma de Mexico, Sisal, Mexico; ^3^ Unidad de Quimica Sisal, Facultad de Quimica, Universidad Nacional Autonoma de Mexico, Sisal, Mexico; ^4^ Laboratorio Nacional de Resiliencia Costera (LANRESC), Laboratorios Nacionales, CONACYT, Mexico City, Mexico; ^5^ Laboratorio de Ecofisiologia de Organismos Acuaticos, Departamento de Biotecnologia Marina, Centro de Investigacion Cientifica y de Educacion Superior de Ensenada (CICESE), Ensenada, Mexico; ^6^ Instituto de Acuicultura, Universidad Austral de Chile, Puerto Montt, Chile; ^7^ Centro de Investigación de Dinámica de Ecosistemas Marinos de Altas Latitudes (IDEAL), Valdivia, Chile; ^8^ Instituto Milenio Biodiversidad de Ecosistemas Antárticos y Subantárticos (BASE), Valdivia, Chile

**Keywords:** *Callinectes sapidus*, dissolved oxygen, thermal changes, routine metabolism, physiological indicators

## Abstract

Dissolved oxygen (DO) and water temperature vary in coastal environments. In tropical regions, the ability of aquatic ectotherms to cope with hypoxia and high-temperature interactive effects is fundamental for their survival. The mechanisms underlying both hypoxia and thermal tolerance are known to be interconnected, therefore, the idea of cross-tolerance between both environmental stressors has been put forward. We investigated the combined role of hypoxia and temperature changes on the physiological responses of blue crab *Callinectes sapidus* living in the southern Gulf of Mexico. We measured oxygen consumption, plasmatic biochemical indicators, total hemocyte count (THC), and antioxidant activity biomarkers in muscle and gill tissues of blue crab acclimated to moderate hypoxia or normoxia and exposed to a thermal fluctuation or a constant temperature, the former including a temperature beyond the optimum range. Animals recovered their routine metabolic rate (RMR) after experiencing thermal stress in normoxia, reflecting physiological plasticity to temperature changes. In hypoxia, the effect of increasing temperature was modulated as reflected in the RMR and plasmatic biochemical indicators concentration, and the THC did not suggest significant alterations in the health status. In both DO, the antioxidant defense system was active against oxidative (OX) damage to lipids and proteins. However, hypoxia was associated with an increase in the amelioration of OX damage. These results show that *C. sapidus* can modulate its thermal response in a stringent dependency with DO, supporting the idea of local acclimatization to tropical conditions, and providing insights into its potential as invasive species.

## 1 Introduction

Hypoxia can originate in marine and freshwater environments, and its occurrence is constrained by hydrological processes, climate, and human activities (Zillén et al., 2008; Levin et al., 2009; Zhang et al., 2010). Episodic hypoxia can occur in estuarine and brackish shallow environments on a diel basis (diel-cycling hypoxia) since nighttime metabolic rates exceed oxygen resupply from the atmosphere (Smith and Able, 2003; Diaz and Rosenberg, 2008; Andersen et al., 2017; Borowiec et al., 2018). In shallow coastal areas, short-term hypoxia is provoked by upwelling events following seasonal patterns, and also by anthropogenic nutrient loading related to freshwater influx (Grantham et al., 2004).

Most benthic species living in fluctuating dissolved oxygen (DO) regimes possess different degrees of regulatory capacity (McMahon, 2001). In crustaceans, the primary response to hypoxia is increasing ventilation of the branchial chambers to maintain their metabolic rate—often measured as oxygen consumption rate (Cech and Brauner, 2011)—up to a critical oxygen level (Pc), that is, the concentration beneath which metabolic rate becomes oxygen dependent (Seibel et al., 2021). Below the Pc other mechanisms operate in response to hypoxic stress. Some of these responses include increasing hemocyanin concentration to improve hemolymph oxygen carrying capacity as seen in *Cancer magister* (Head, 2010), hypoxia-induced bradycardia to reduce overall cardiac work as reported in the grass shrimp *Palaemonetes pugio* (Guadagnoli et al., 2007; 2011), and metabolic rate depression (Sokolova et al., 2012). Overall, this suite of physiological adjustments aims at minimizing the disruption on the balance between energy supply and demand, defined as energy metabolism, until the organisms reach a lethal range of DO (Sokolova et al., 2012).

Based on the influence of temperature on energy metabolism, the oxygen-and capacity-limited thermal tolerance (OCLTT) hypothesis (Pörtner, 2002; 2010; Pörtner et al., 2006) highlights the interdependence of hypoxic and thermal stress responses. Organismal thermal tolerance limits are reached at temperatures where the cardiorespiratory system fails to meet tissue metabolic demands (Pörtner and Farrell, 2008). In the optimum temperature range, the aerobic capacity covers the energy demands of maintenance and major functions. In moderate thermal stress (the *pejus* range), the aerobic capacity is still positive but diminished due to an elevated routine metabolic rate (RMR)—the average metabolic rate of a postabsorptive ectotherm with free movement at a defined temperature—and the acclimation in protection capacity triggers key role mechanisms to maintain homeostasis (Pörtner, 2010; Sokolova et al., 2012). When thermal stress is extreme (the *pessimum* range), survival becomes time dependent, which implies a functional association between tolerance to temperature and hypoxia at all biological levels.

The effects of chronic hypoxia and thermal stress in crustaceans have been evaluated on physiological and biochemical traits. Previous studies have demonstrated that crustaceans may exploit glycolysis for energy production resulting in lactate accumulation (Aparicio-Simón et al., 2018), while an increment in reactive oxygen species (ROS) production is expected at mitochondrial level as a by-product of higher cell respiration when temperature increases (Romero et al., 2013) or DO decreases (Trasviña-Arenas et al., 2013). In gills, the activities of antioxidant enzymes increase after hypoxia exposure, returning to normal levels in normoxia (e.g., *L. vannamei* (García-Triana et al., 2010; Trasviña-Arenas et al., 2013)); there is evidence that elevated levels of antioxidant defense enzymes during hypoxia might occur as an adaptive strategy in fluctuating environments in preparation for oxidative stress during reoxygenation (Hermes-Lima et al., 2015). Furthermore, common pollution biomarkers, including the activities of cholinesterases and carboxylesterases, might provide insights on physiological condition at given temperature or DO regime. For instance, some authors report evident seasonal fluctuation in acetylcholinesterase (AChE) (e.g., Menezes et al., 2006), a key enzyme in cholinergic signal transmission in the sensory and neuromuscular systems that therefore appears to be likely related to animals’ locomotor activity (Anita et al., 2017). Moreover, carboxylesterases (CbE), a multifunctional group of enzymes relevant in lipid synthesis and decomposition and signal transmembrane transduction (Zang et al., 2020), are involved in the metabolism of endogenous compounds (Nos et al., 2021), and thermal stress can compromise their capability to face environmental chemical stressors (Saiz et al., 2022).

In terms of adaptation, the relationship between the mechanisms underlying thermal and hypoxia tolerance put forward the idea that acclimation to one stressor might enhance tolerance to another one (i.e. there is cross-tolerance) (Burleson and Silva, 2011; McBryan et al., 2016). Moreover, the ability of aquatic ectotherms to tolerate simultaneously hypoxia and elevated temperatures—likely to occur under climate change scenarios—together with all niche elements determine ecosystem stability (Masson-Delmotte et al., 2019). Nevertheless, scarce information exists about whether hypoxic acclimation improves thermal performance in crustaceans, and *vice versa*.

Among the biologically and commercially important decapod crustaceans, blue crab *Callinectes sapidus*—a keystone species (Glaspie et al., 2020)—has one of the broadest latitudinal native distribution in coastlines and adjacent wetlands on the western Atlantic where some of the species distribution spots coincide with hypoxic zones (Rabalais et al., 2007; Johnson, 2015; Seitz, 2020). The physiology of blue crab in tropical conditions remains relatively unexplored (Ángeles-González et al., 2020). However, García-Rueda et al. (2021) reported lower and upper thermal window limits of 11°C and 42°C, respectively; the highest thermal metabolic scope (TMS, a proxy for the aerobic window) at 32°C, and the highest hypoxia tolerance from 29°C to 34°C with a 50% reduction in RMR at ∼20% air saturation. Nonetheless, considering that blue crab in tropical conditions are close to their upper thermal limits, in warming scenarios where higher temperatures and more severe hypoxia are likely to occur (Glenn et al., 2015; Murray-Tortarolo, 2021), they are thought to be susceptible to future strong oxygen-temperature interactions.

We conducted lab-based experiments to evaluate the responses of oxygen consumption and biochemical indicators of blue crab from the southern Gulf of Mexico after being acclimated to different DO and thermal regimes to provide information about the interactive effects of both environmental stressors in animals from the tropics. For this purpose, we implemented two different thermal regimes (i.e. fluctuating and constant) under long-term moderate hypoxia and normoxia to test the hypothesis of cross-tolerance on routine metabolism.

## 2 Materials and methods

### 2.1 Animal collection, maintenance, and experimental setup

Animal ethics approval was not required for the use of blue crab in research in Mexico, in accordance with national guidelines. However, for all experiments and animal husbandry, we used established criteria for good management practices for aquatic animals based on the norms of conduct of Universidad Nacional Autónoma de México (UNAM) and Centro de Investigación Científica y de Educación Superior de Ensenada.

Adult blue crab (*n* = 97; mean mass ± standard deviation = 189.35 ± 36.85 g) were collected from wild population close to Sisal, Yucatán, México (21 09’ 55’’ N and 90 01’ 50” W), using artisanal fishing traps. Females bearing eggs and individuals with conspicuous external lesions were excluded. Organisms were transported to the Laboratory of Applied Ecophysiology (Faculty of Sciences) at UNAM, Sisal, Yucatan. Blue crab were subjected to a conditioning period for 2 weeks to ensure that all had a similar recent thermal history (Vinagre et al., 2016) and allow them to fully recover from capture and handling in 3000 L tanks (maximum 30 organisms per tank) filled with flow-through natural filtered (20 µm) seawater (35–37 PSU) at 26°C ± 1°C and ≥80% air saturation, under a 12 h:12 h light/dark photoperiod. Organisms were fed small pieces of squid or fish twice a day, and feces and food residues were removed daily from the cages and tanks with a siphon. At the end of the laboratory conditioning period, epibionts from the crab carapace and abdomen were removed.

**FIGURE 1:**
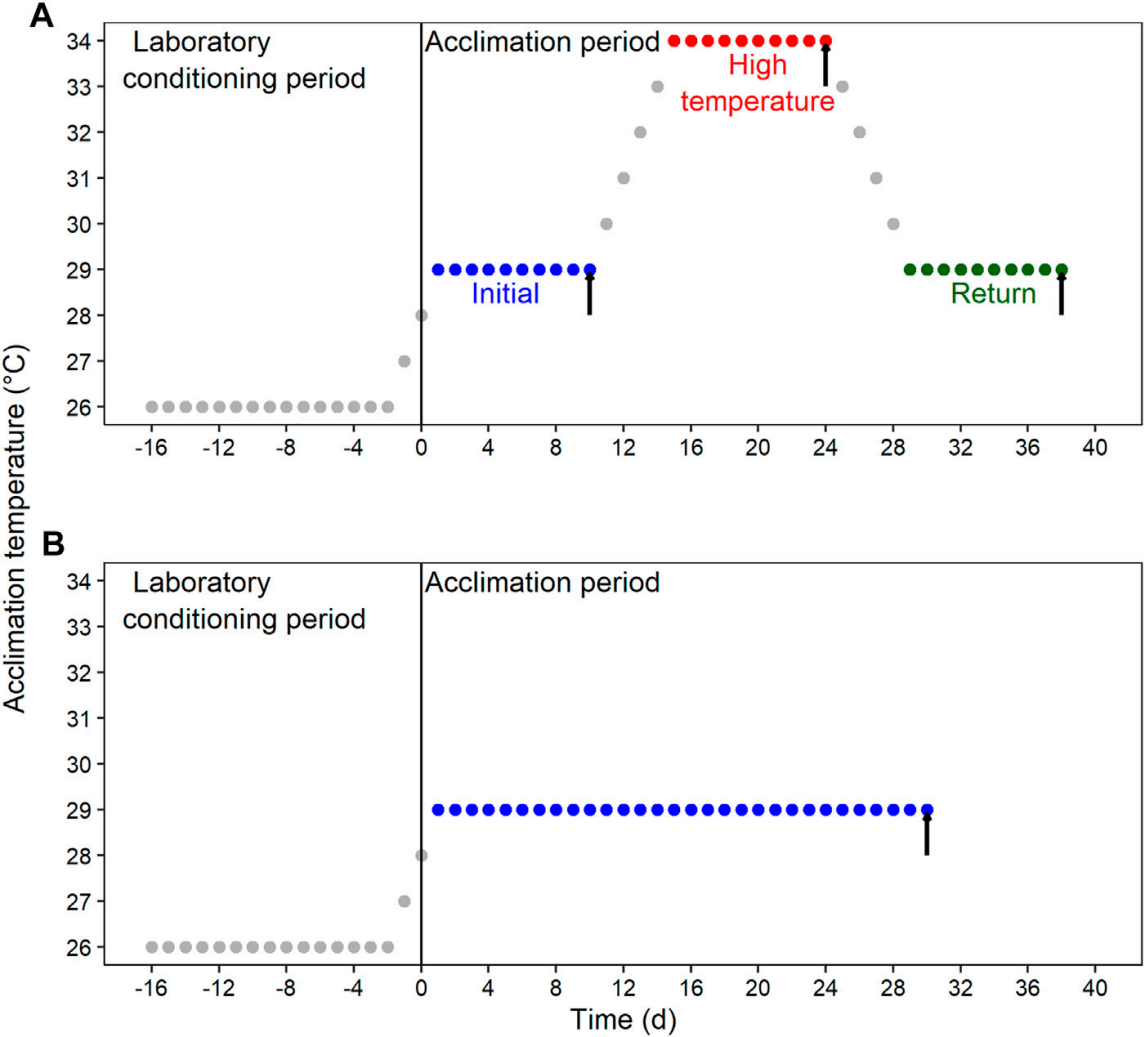
Schematic representation of the experimental design for the laboratory conditioning period and acclimation of *Callinectes sapidus*. **(A)** Fluctuating condition with an initial section at 29°C, followed by a high-temperature section at 34°C, and a return section at 29°C. **(B)** Constant condition at 29°C. Both thermal regimes were implemented in normoxia (≥80% air saturation) and moderated hypoxia (45%–50% air saturation). Black vertical arrows indicate the sampling time for respiratory and biochemical variables.

After the laboratory conditioning period, blue crab were transferred to Multi-stressor units (Aquabiotech INC, Coaticook, Quebec, Canada) that allow the control for several environmental variables. Animals were randomly distributed in the “habitat racks” and subjected to four acclimation conditions resulting from the combination of DO and thermal regime. The first DO was normoxia at ≥80% air saturation, and the second one was moderate hypoxia at 45%–50% air saturation. Thermal acclimation was either fluctuant or constant. Therefore, the resulting four acclimation conditions were: 1) thermal fluctuation in normoxia; 2) thermal fluctuation in moderate hypoxia; 3) constant temperature in normoxia; and 4) constant temperature in moderate hypoxia. The thermal fluctuation consisted of three consecutive 10-day (d) sections, each one followed by a sampling day: 1) initial section at 29°C (sampling at 10d); 2) high-temperature section at 34°C (sampling at 24d); and 3) return section at 29°C (sampling at 38d) (Figure 1A). The constant temperature lasted for 30d at 29°C (Figure 1B).

The required temperature changes were made at a rate of ∼1°C d^−1^ to reduce the risk of inducing additional thermal stress. Salinity, feeding, individualization cages cleaning, and photoperiod were maintained as described above for the laboratory conditioning period. Because seawater circulating through the Multi-stressor systems is partially recycled, we monitored nutrients (total ammonia, nitrite, and nitrate) periodically to verify that they were maintained under the safe range for the species. A group of blue crab was randomly selected at each sampling time to perform the measurements, and animals were fasted for 24 h to ensure they were in a post-absorptive condition.

### 2.2 Organismal level response: Routine oxygen consumption measurements

Oxygen consumption rate was individually measured using flow-through respirometry, a method that allows to measure oxygen change from a controlled, continuous flow of water through the respirometer. This feature is especially convenient to maintain water inflow with a stable dissolved oxygen (DO) in the respirometric chambers during measurements in hypoxia. All the measurements were done in a semi-dark environment and in the absence of external stimuli. To record DO data, respirometric chambers were connected to a pair (entrance and exit) of flow-through oxygen sensors that were coupled by a fiber cable to a Witrox 4 instrument (Loligo Systems, Viborg, Denmark). Before measurements, all sensors were calibrated for each experimental temperature, using air saturated seawater and an oxygen-free solution with sodium sulfite (0.1 g in 10 ml^−1^ of seawater; 0% OD); the seawater flowrate in each chamber was standardized using a flowmeter. For measurements in hypoxia, once the system reached the testing temperature, DO was reduced using gaseous nitrogen injection directly in the seawater reservoir to 45%–50% air saturation, and animals were placed into the respirometric chambers only when experimental conditions were stable.

Respirometric trials lasted 36 h. An empty chamber (without organism) was used as control to estimate and correct background respiration in each trial. Blue crab were weighed at the end of the experiment, and oxygen consumption was calculated using the following equation:

$$MO_{2=}\frac{{\left(([O\right.}_{2\;mg/l}]I{-[O}_{2\;mg/l}]Out)\cdot \;F\;)\;}{WW}$$

Where ${\left[O\right.}_{2\;mg/l}]I$ and ${\left[O\right.}_{2\;mg/l}]Out$ represent DO at the input and output of each chamber; F is the flux in L h^−1^; and WW the wet weight of each animal. MO_2_ of the control chamber was subtracted from each calculated value and the final rate was expressed as mgO_2_ g^−1^ h^−1^.

### 2.3 Cellular level response: Hemolymph and oxidative stress indicator analysis

Plasmatic biochemical indicators, total hemocyte count (THC), and oxidative stress indicators were quantified from each experimental treatment after RMR measurements. Plasmatic biochemical indicators included total proteins (PRO), cholesterol (CHO), acylglycerides (ACY), glucose (GLU) and lactate (LAC). Antioxidant defense system (ADS) markers–catalase (CAT), glutathione S-transferase (GST), superoxide dismutase (SOD), and total glutathione levels (T.GSH)—and physiological condition biomarkers–activities of acetylcholinesterase (AChE) and carboxylesterase (CbE) –, as well as oxidative indicators (OX)—protein carbonylation (PO) and lipid peroxidation (LPO) –, were analyzed in muscle and gill tissue.

We obtained blue crab hemolymph from the swimming leg coxa with a precooled disposable insulin syringe impregnated internally with anticoagulant solution SIC-EDTA (450 mM NaCl; 10 mM KCl; 10 mM HEPES; 10 mM EDTA). A sample of 10 µl of hemolymph from each organism was processed immediately after extraction to determine the THC using TC10 counting slides with dual chambers. Readings were performed with a TC10™ automated cell counter (Bio-Rad INC, Hercules, California, United States). For plasmatic biochemical indicators quantification, 100 µl of hemolymph from each organism was homogenized with SIC-EDTA (1:2 ratio) in individual Eppendorf tubes and refrigerated until analysis within the next two hours. The samples were centrifuged (800 g, 4°C for 5 min, Bio-Rad INC, Hercules, California, United States) to obtain plasma as supernatant. CHO, ACY, GLU, and LAC were quantified by colorimetric analysis in microplate using 10 µl of plasma and 200 µl of enzyme chromogen reagent in commercial kits (ELITech Group, Puteaux, Paris, France) following the manufacturer’s instructions. PRO were determined following the Bradford method (1976). Concentration (mg ml^−1^) was estimated using the absorbance value of each sample, values obtained from the linear regression analysis of the standard absorbance curve (intercept and slope), and the value of the dilution factor:

$$Concentration\;\left(\frac{mg}{ml}\right)=\frac{\left(sample\;absorbance-intercept\right)\;}{slope}\cdot dilution\;factor$$

Blue crab were sacrificed immediately after taking hemolymph samples. After that, a portion of locomotor muscle and gill tissue were separated on ice, snap-frozen in liquid nitrogen, and then stored at −80°C until further analysis. Samples were homogenized with TRIS 0.05 M pH 7.4 at 100 mg tissue ml^−1^ using a Potter-Elvehjem homogenizer. The sample homogenate was divided in Eppendorf tubes, each containing enough volume for duplicate assays of AChE (Ellman et al., 1961 adapted by Rodríguez-Fuentes et al., 2008), CbE (Hosokawa and Satoh, 2002), CAT (Góth, 1991 modified by Hadwan and Abed, 2016), GST (Habig and Jakoby, 1981), SOD (Sigma-Aldrich assay kit 19160), PO (Mesquita et al., 2014), LPO (Sigma-Aldrich PeroxiDetect Kit), and T.GSH (Sigma-Aldrich Glutathione Assay Kit CS0260). Prior quantifications of AChE, CbE, SOD, CAT, and GST, muscle and gill homogenates were centrifuged (10,000 g, 4°C for 5 min, Bio-Rad INC, Hercules, California, United States) and the supernatant was separated for analysis. Proteins were analyzed in the supernatant following Bradford (1976) to standardize all enzyme activities in activity unit (U) mg^−1^ protein.

### 2.4 Data analyses

We used two fixed explanatory factors. The first factor was dissolved oxygen (DO) with two levels: normoxia and hypoxia. The second factor was sampling time, which represented the combination of thermal regime (fluctuant or constant) and time, consisting of four different levels: 1) 10d (i.e., 10d at 29°C); 2) 24d (i.e., 10d at 29°C + 10d at 34°C); and 3) 38d (i.e., 10d at 29°C + 10d at 34°C + 10 days at 29°C) along the thermal fluctuation and 4) constant temperature at 29°C lasting 30d (Figure 1).

We analyzed the differences between routine metabolic rate (RMR) using a Linear Mixed-effects Model and tested the significance of including replicates in the experimental setup as a random factor and variance structure that accounts for changes in error-variance due to one or both fixed factors using the likelihood ratio and the Akaike Information Criterion (AIC). We visually inspected model standardized residuals (i.e., residuals *versus* fitted values and quantile-quantile plots) for outliers, normality, and variance homogeneity, and we applied pairwise *post hoc* comparisons between sampling times for each DO based on the model estimated parameters.

We performed a two-way ANOVA to assess the effects of DO and sampling time on total hemocyte count (THC). Here again, residuals were visually inspected. We also performed normality (Shapiro-Wilk test) and variance homogeneity (Levene’s test) tests on the model residuals. A Fisher Least Significant Difference (LSD) *post hoc* test was conducted.

The response of energetic and stress indicators was evaluated separately for 1) plasmatic biochemical indicators concentrations, 2) antioxidant defense system (ADS) and oxidative (OX) damage biomarkers, and 3) physiological condition biomarkers. All variables were Log(x+1) transformed and normalized (centered on their means) previous to analysis. A permutational analysis of variance (PERMANOVA) was used to test the hypothesis of statistical differences between experimental groups. The homogeneity of the multivariate dispersion in the Euclidean space was previously tested with a multivariate analogue to Levene’s test (PERMDISP), using distances to centroids. Similarity percentages (SIMPER) analysis was also used to determine which dependent variables contributed most to the observed pattern of separation between experimental groups. All procedures were based on 9999 permutations under the reduced models (Anderson, 2006). Plasmatic biochemical indicators concentrations and ADS and OX damage biomarkers were visualized by means of a Principal Coordinate Analysis (PCoA) applied on the resemble matrixes of Euclidean distances. Furthermore, linear models using Generalized Least Squares (GLS) were used to analyze the patterns of univariate responses. We performed a procedure through restricted maximum likelihood to test different weights or within-group heteroscedasticity structure terms (allow errors to have unequal variances), and we selected the most informative model based on visual inspection of Pearson’s residuals and AIC (Montgomery and Peck, 1992; Zuur et al., 2007). We conducted two-way ANOVA analyses and Tukey *post hoc* comparisons for statistically significant ANOVA results.

We conducted statistical analyses using R Studio and PRIMER 7. R package nlme (Phineiro et al., 2022) was used to adjust Linear Mixed-effects Model and GLS models; package vegan (Oksanen et al., 2022) was used to conduct PCoA analyses; and ggplot2 (Wickham, 2016) was used to generate graphic visualizations. Data are shown as mean value ± standard deviation.

## 3 Results

### 3.1 Routine metabolic rates (RMR)

**FIGURE 2:**
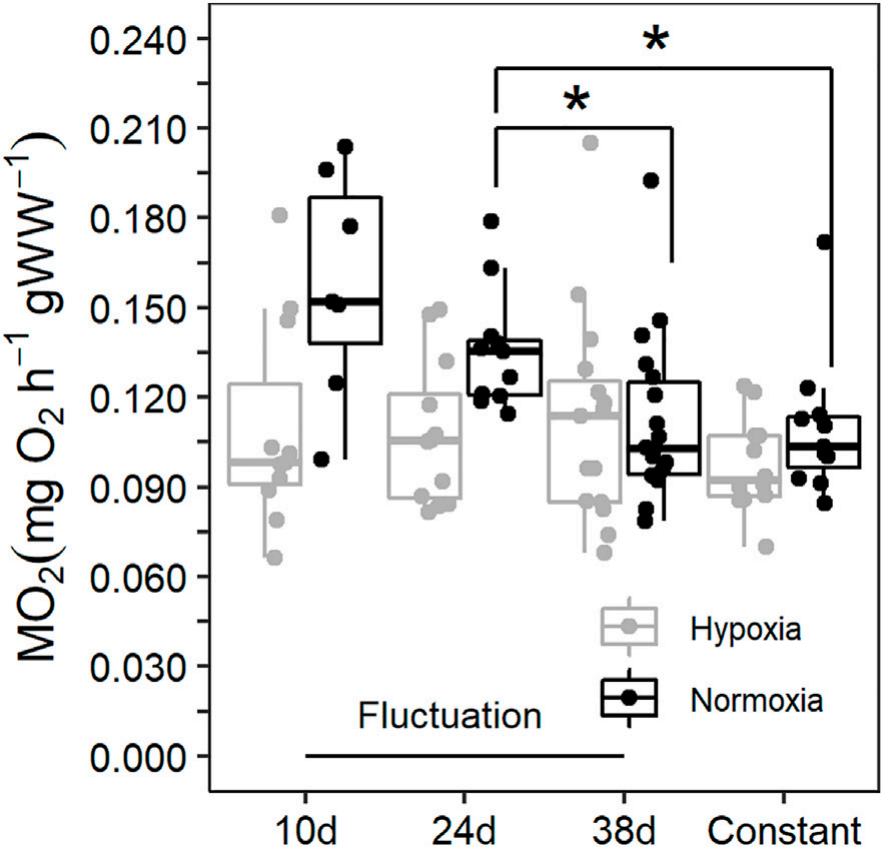
Routine metabolic rate (RMR) of *Callinectes sapidus* acclimated to a thermal fluctuation or a constant temperature in normoxia (≥80% air saturation) and moderate hypoxia (∼45% air saturation). Sampling times along the fluctuation are 10d at 29°C (*n* = 7 in normoxia and 11 in hypoxia), 24d at 34°C (*n* = 11 in normoxia and 12 in hypoxia), and 38d at 29°C (*n* = 18 in normoxia and 15 in hypoxia). The constant temperature lasted for 30d (*n* = 11 in normoxia and 12 in hypoxia). The asterisks symbols refer to statistically significant difference at *p* ≤ 0.05.

The most informative model included the random factor associated to the replication of the acclimation conditions (AIC = −360, L. Ratio = 15.11, *p* < 0.001 compared to the reduced model without the random factor), a variance structure accounting for changes in error-variance between regimes (AIC = −362, L. Ratio = 8.90, *p* < 0.05 compared to the reduced model without the variance structure), and the interaction between dissolved oxygen (DO) and sampling time (AIC = −423, L. Ratio = 11.75, *p* < 0.01 compared to the reduced model lacking the interaction term). Our results showed that the thermal fluctuation provoked changes across sampling times in the RMR of blue crab acclimated to normoxia (Figure 2). The highest RMR was observed in blue crab after the first 10d at 29°C, followed by progressive reductions after they experienced 34°C (24d, *p* < 0.01) and then returned to 29°C (38d, *p* < 0.001). Similar values were reached at the end of the thermal fluctuation compared to the constant temperature (0.11 ± 0.03 mgO_2_ g^−1^ h^−1^ and 0.11 ± 0.02 mgO_2_ g^−1^ h^−1^, respectively; *p* = 0.223). In blue crab acclimated to hypoxia, the RMR did not change across sampling times in the thermal fluctuation (Figure 2; *p* > 0.05 for all comparisons), and no significant differences were observed in the RMR of blue crab maintained in constant temperature (0.1 ± 0.02 mgO_2_ g^−1^ h^−1^) compared to those evaluated at each sampling time during fluctuation (*p* > 0.05 for all comparisons) (Figure 2).

### 3.2 Total hemocytes count (THC)

TCH was significantly affected by DO (ANOVA: F-value = 4.4; *p* < 0.05). Sampling time and its interaction with DO were unsignificant (F-value = 2.1; *p* = 0.122, and F-value = 1.3; *p* = 0.284, respectively). However, significant differences were only detected for the first 10 d in the thermal fluctuation (*p* < 0.05) (Supplementary Table S1).

### 3.3 Plasmatic biochemical indicators

**FIGURE 3:**
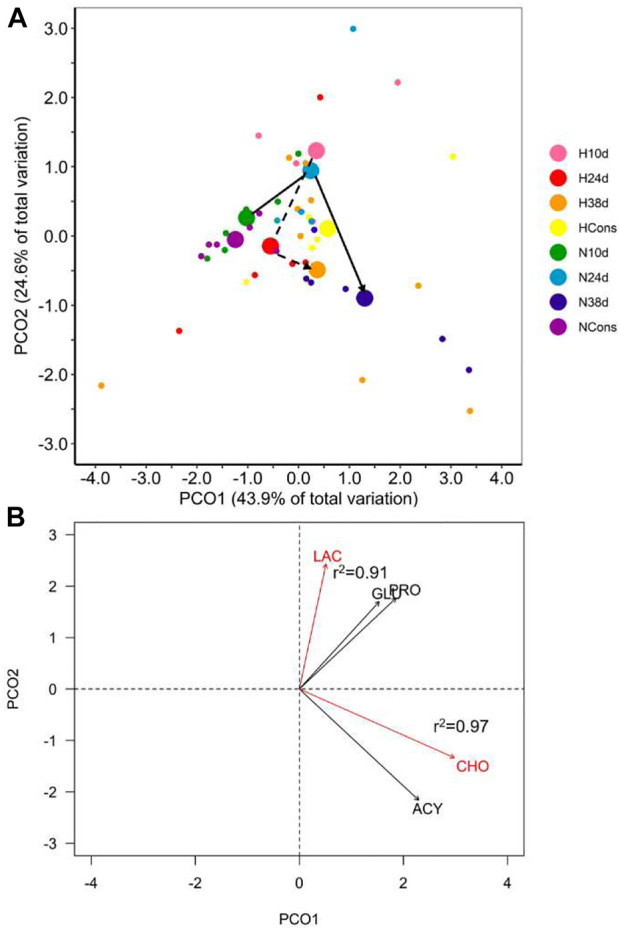
Plasmatic biochemical indicators in blue crab hemolymph. **(A)** Principal coordinate analysis (PCoA) of centroids and individual points per DO (N = normoxia; H = hypoxia) and sampling time (10d, 24d, and 38d in the thermal fluctuation, and the constant temperature). The lines follow the centroid trajectories (solid line = normoxia; dashed line = hypoxia) along the fluctuation. DO and sampling time combinations are referred as N10d (*n* = 6), N24d (*n* = 4), N38d (*n* = 6), NCons (*n* = 6), H10d (*n* = 4), H24d (*n* = 5), H38d (*n* = 9), and HCons (*n* = 5). **(B)** Biplot of biochemical indicators. Vectors in red and Pearson correlation values correspond to the most correlated variables with PCO1 and PCO2. LAC = lactate, GLU = glucose, PRO = total proteins, CHO = cholesterol, ACY = acylglycerides.

The interaction between dissolved oxygen (DO) and sampling time was significant (Table 1). This result indicates that the alteration in plasmatic biochemical indicators caused by temperature changes depended on DO. This is supported by differential distribution of sampling times centroids by DO in the PCoA for the distance matrix (Figure 3A). Cholesterol (CHO) and lactate (LAC) were the most correlated variables with PCO1 and PCO2, respectively, being the most influential ones in groups ordination (Figure 3B). Pairwise multivariate comparisons among sampling times at each DO were significant for all contrasts only in normoxia (Table 2), and the SIMPER analysis revealed that peaks in LAC, glucose (GLU) and lipids (acylglycerides (ACY) and CHO) levels at 10d, 24d and 38d in the fluctuation, respectively, provoked these differences (Supplementary Figure S1, Table 2).

**TABLE 1:** Permutational analysis of variance (PERMANOVA) of the effect of DO and sampling time on blue crab. A = plasmatic biochemical indicators, B = ADS and OX damage biomarkers in muscle, C = ADS and OX damage biomarkers in gill, D = physiological condition biomarkers in muscle, and E = physiological condition biomarkers in gill. The probabilities associated at each F-ratio were obtained with 9999 permutations of residuals under a reduced model.

A				
Source	df	MS	*F*	*p*
Dissolved oxygen (DO)	1	3.52	0.90	0.460
Sampling time (Sa)	3	12.64	3.22	0.001
DO x Sa	3	11.07	2.82	0.003
Res	37	3.93		
Total	44			
B				
Source	df	MS	*F*	*p*
Dissolved oxygen (DO)	1	15.86	2.94	0.013
Sampling time (Sa)	3	10.72	1.99	0.015
DO x Sa	3	5.26	0.98	0.477
Res	35	5.38		
Total	42			
C				
Source	df	MS	*F*	*p*
Dissolved oxygen (DO)	1	17.73	3.49	0.008
Sampling time (Sa)	3	9.96	1.96	0.021
DO x Sa	3	9.11	1.79	0.042
Res	36	5.08		
Total	43			
D				
Source	df	MS	*F*	*p*
Dissolved oxygen (DO)	1	11.79	9.76	0.0005
Sampling time (Sa)	3	7.05	5.83	0.0002
DO x Sa	3	3.44	2.85	0.0117
Res	37	3.44		
Total	44	1.21		
E				
Source	df	MS	*F*	*p*
Dissolved oxygen (DO)	1	15.79	11.78	0.0001
Sampling time (Sa)	3	5.04	3.76	0.0033
DO x Sa	3	2.28	1.71	0.1393
Res	36	1.34		
Total	43			

**TABLE 2:** Results of pair-wise comparisons among sampling times at each DO based on the Euclidean distance matrix. Results of the SIMPER analysis are included for significant comparisons. A = plasmatic biochemical indicators, B = ADS and OX damage biomarkers in muscle, C = ADS and OX damage biomarkers in gill, D = physiological condition biomarkers in muscle, E = physiological condition biomarkers in gill, Var. = variable, and %Cont. = contribution of individual variables to the overall Euclidean dissimilarity (cut-off of 70%). The probabilities associated at each F-ratio were obtained with 9999 permutations of residuals under a reduced model.

	Normoxia				Hypoxia			
	Pair-wise test		SIMPER		Pair-wise test		SIMPER	
Groups	*t*	*p*	Var.	% Cont.	*t*	*p*	Var.	% Cont.
A								
10d, 24d	2.471	0.005	GLU	46.43	1.413	0.156	−	−
			LAC	29.55				
10d, 38d	3.204	0.002	ACY	44	1.296	0.166	−	−
			CHO	37.09				
10d, Cons	2.368	0.004	LAC	45	1.855	0.05	−	−
			PRO	30				
24d, 38d	2.261	0.013	ACY	46	0.741	0.689	−	−
			GLU	18.45				
			CHO	17				
24d, Cons	2.896	0.004	GLU	44.7	1.453	0.057	−	−
			LAC	27.34				
38d, Cons	3.594	0.002	CHO	51.5	1.014	0.444	−	−
			ACY	39.3				
B								
10d, 24d	1.593	0.04	CAT	32.52	0.724	0.792	−	−
			LPO	30.98				
			SOD	13.78				
10d, 38d	2.705	0.006	CAT	28.81	1.042	0.369	−	−
			SOD	28.21				
			T.GSH	13.47				
10d, Cons	2.047	0.023	PO	48.49	1.201	0.221	−	−
			SOD	19.2				
			CAT	12.58				
24d, 38d	1.467	0.122	−	−	0.901	0.568	−	−
24d, Cons	1.049	0.368	−	−	0.664	0.784	−	−
38d, Cons	1.094	0.306	−	−	1.02	0.429		
C								
10d, 24d	1.121	0.286	−	−	1.008	0.371	−	−
10d, 38d	2.198	0.003	PO	35.41	0.769	0.721	−	−
			CAT	15.81				
			LPO	13.34				
			T.GSH	13.02				
10d, Cons	1.337	0.134	−	−	1.323	0.172	−	−
24d, 38d	1.708	0.009	LPO	39.64	0.592	0.896		
			PO	33.38				
24d, Cons	0.772	0.678	−	−	1.176	0.232	−	−
38d, Cons	1.977	0.006	PO	54.32	1.051	0.354	-	-
			SOD	14.31				
			LPO	12.95				
D								
10d, 24d	1.644	0.071	−	−	1.356	0.225	−	−
10d, 38d	2.968	0.003	AChE	60.8	1.357	0.202	-	-
			CbE	39.2				
10d, Cons	1.234	0.216	−	−	1.591	0.125	−	−
24d, 38d	4.372	0.004	AChE	87.77	0.869	0.477	−	−
24d, Cons	2.555	0.018	AChE	69.03	1.028	0.404	−	−
			CbE	30.97				
38d, Cons	3.952	0.002	AChE	79.14	0.511	0.768	−	−
E								
10d, 24d	2.02	0.079	−	−	2.616	0.006	CbE	74.61
10d, 38d	4.849	0.002	CbE	58.67	1.195	0.254	−	−
			AChE	41.33				
10d, Cons	1.504	0.129	−	−	1.16	0.31	−	−
24d, 38d	1.188	0.313	−	−	0.762	0.573	−	−
24d, Cons	1.412	0.167	−	−	0.676	0.752	−	−
38d, Cons	4.117	0.002	AChE	73.52	0.066	0.994	−	−

No statistical differences were detected in terms of multivariate dispersion of the groups data (combinations of DO and sampling time) from the centroids (PERMDISP, *p* = 0.066). Therefore, the dispersion effect is not driving the existing differences among groups in normoxia nor the lack of differences among them in hypoxia.

### 3.4 Oxidative stress and physiological condition biomarkers

#### 3.4.1 Muscle

**FIGURE 4:**
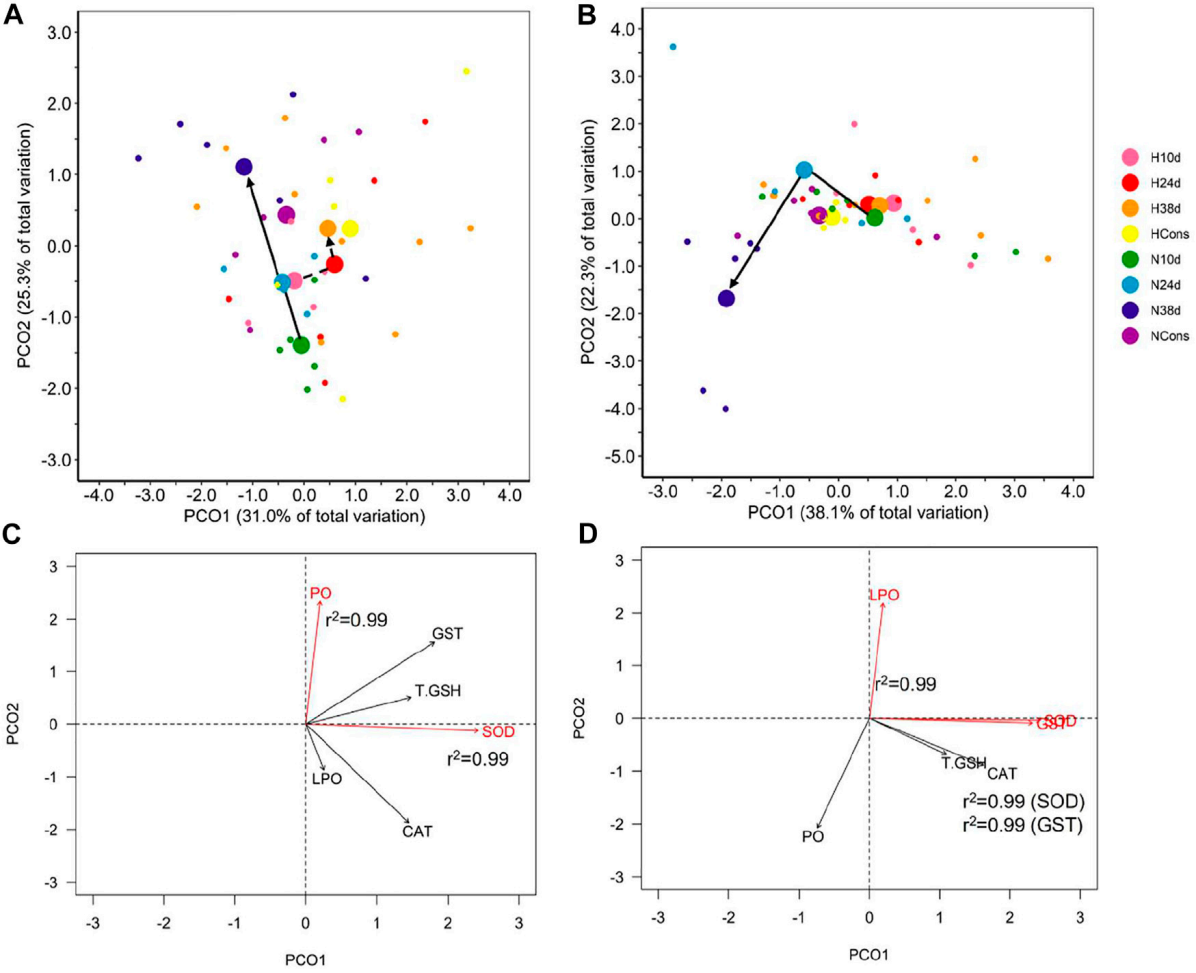
Antioxidant defense system and oxidative damage indicators in tissues. Principal coordinate analysis (PCoA) of centroids and individual points per DO (N = normoxia; H = hypoxia) and sampling time (10d, 24d, and 38d in the thermal fluctuation, and the constant temperature) for muscle **(A)** and gill **(B)**. The lines follow the centroid trajectories (solid line = normoxia; dashed line = hypoxia) along the fluctuation. DO and sampling time combinations are referred as N10d (*n* = 6), N24d (*n* = 4), N38d (*n* = 6), NCons (*n* = 6), H10d (*n* = 4), H24d (*n* = 5), H38d (*n* = 9), and HCons (*n* = 5). Biplot of biochemical indicators for muscle **(C)** and gills **(D)**. Vectors in red and Pearson correlation values correspond to the most correlated variables with PCO1 and PCO2. LPO = lipid peroxidation, PO = protein carbonylation, CAT = catalase, SOD = superoxide dismutase, T.GSH = total glutathione, and GST = glutathione S-transferase.

A significant effect of sampling time and DO on the antioxidant defense system (ADS) and oxidative (OX) damage in muscle of blue crab was observed, while the interaction between both factors was not significant (Table 1). Therefore, temperature modulated the ADS and the OX damage in muscle of blue crab acclimated to normoxia and hypoxia following a similar pattern (Figure 4A). Superoxide dismutase (SOD) and protein carbonylation (PO) were the most correlated variables with PCO1 and PCO2, respectively, being the most influential ones in groups ordination (Figure 4C). Significant pairwise comparisons among sampling times occurred only between 10d and the rest of sampling times in normoxia, which is associated to higher levels of catalase (CAT) and SOD, and lower levels of PO according to the SIMPER analysis (Supplementary Figures S2, S3, Table 2). However, significant differences in dispersion were observed in terms of multivariate dispersion of the groups data (combinations of DO and sampling time; PERMDISP, *p* = 0.024), indicating that the lack of differences between sampling times was associated with the variability in concentrations.

Differences in the activity of carboxylesterase (CbE) and acetylcholinesterase (AChE) in muscle of blue crab were attributed to sampling time, DO, and the interaction between both factors (Table 1). Significant pairwise comparisons among sampling times were found only in normoxia between 38d and the rest of sampling times, as well as between 24d and the constant temperature (Table 2). The SIMPER analysis revealed that lower and higher levels of AChE at 38d and 24d, respectively, were responsible for these differences (Supplementary Figure S4, Table 2).

No statistical differences were detected in the physiological condition indicators data (PERMDISP, *p* = 0.145). Therefore, the dispersion effect is not driving the existing differences among groups in normoxia nor the lack of differences among them in hypoxia.

#### 3.4.2 Gills

A significant effect of sampling time, DO, and the interaction between these factors was observed on the ADS and the OX damage in gill of blue crab (Table 1). This result indicates that the alteration in these indicators caused by temperature changes depended on DO (Figure 4B). SOD and glutathione S-transferase (GST) were the most correlated variables with PCO1, while LPO was the most correlated variable with PCO2, being the most influential ones in groups ordination (Figure 4D). The PCoA of centroids for the distance matrix showed that the differences among sampling times increased in normoxia compared to hypoxia, particularly between 24d (34°C) and 38d (29°C) in the thermal fluctuation (Figure 4B). Pairwise test showed that statistically significant differences existed only between 38d (29°C) and the rest of sampling times in normoxia due to higher levels of PO and lower levels of lipid peroxidation (LPO) and CAT (Supplementary Figures S2, S3, Table 2).

No statistical differences were detected in terms of multivariate dispersion of the groups data (combinations of DO and sampling time) from the centroids for ADS and OX damage in gill (PERMDISP, *p* = 0.113). Therefore, the dispersion effect is not driving the existing differences among groups in normoxia nor the lack of differences among them in hypoxia.

Statistical differences were found between the activity of CbE and AChE in gills of blue crab of different sampling times and DO, while the interaction term was not significant (Table 1). Significant pairwise comparisons among sampling times were detected between 10d and 24d in hypoxia. In normoxia significant differences occurred between 10d and 38d, as well as between 38d and the constant temperature (Table 2). The SIMPER analysis showed that higher levels of CbE at 10d contributed drove the differences in both DO, whereas lower levels of AChE at 38d were responsible for the differences only in normoxia (Supplemenetary Figure S4, Table 2). However, significant differences in dispersion were observed in the physiological condition indicators data (PERMDISP, *p* = 0.028), indicating that the lack of differences between sampling times was associated with the variability in concentrations.

## 4 Discussion

The results in this study provide a novel insight into the energetic and stress response at organismal and cellular levels in blue crab in tropical conditions subjected to the interaction of two key environmental variables: dissolved oxygen (DO) and temperature.

### 4.1 Energetic response

DO modulated the temperature effect on energy demands of blue crab. In normoxia, changes in temperature provoked alterations in the routine metabolic rate (RMR), whereas chronic moderate hypoxia reduced oxygen delivery, neutralizing the passive effects of temperature fluctuation on RMR. Reducing metabolic rate may allow the blue crab to conserve energy and extend their survival time under hypoxia (Hochachka and Somero, 2002; King et al., 2022).

Despite the alterations in RMR under normoxia along the thermal fluctuation, blue crab had the ability to recover their physiological condition after experiencing a *pejus* temperature (34°C; García-Rueda et al., 2021). Animals that returned to 29°C showed a similar RMR to those maintained at constant 29°C. This result agrees with Jayasundara and Somero (2013), who found that the longjaw mudsucker *Gillichthys mirabilis* showed metabolic rate recovery when temperature returned to a value within the preferred range after experiencing suboptimum high temperatures, reflecting physiological plasticity to temperature changes.

The idea that the reduced oxygen delivery to tissues helped blue crab to cope with thermal stress under hypoxia is supported by attenuation in shifts in dominant hemolymph fuel molecules in hypoxia. Crustaceans use glycogen, proteins, and several lipids as the main metabolic fuels, which are transported to tissues in hemolymph and respond to seasonal changes in temperature (Vinagre et al., 2007; Buckup et al., 2008; Pellegrino et al., 2008). In animals acclimated to normoxia, a hyperglycemic response was observed when they were exposed to 34°C in the thermal fluctuation, indicating that glycogenolysis (glycogen catabolism) was turned ON to release glucose (GLU) that presumably satisfied the metabolic requirements provoked by high temperature. Similar results were obtained in many crustacean species (e.g., Vasudevan and Rajendran, 2021), in which the glycogenolysis pathway is the first mechanism to fuel the physiological process involved to maintain homeostasis under stress conditions. Moreover, significantly higher lipid levels were detected when blue crab returned to 29°C at the end of the thermal fluctuation, which suggested that lipolysis (triglycerides catabolism) was used as a second source of energy after the glycogen-glucose pathway. The primary metabolic reserve in most crustaceans are acylglycerides (ACY), mainly triglycerides (Alava et al., 2007), while and cholesterol (CHO) is a key structural component in cell membrane structure and it serves as a precursor of physiological components including sex and molting hormones (Wouters et al., 2001; Kumar et al., 2018). Therefore, a possible hypothesis is that the peaks of these lipids in crab hemolymph were due to metabolic debt, membrane repair mechanism, and molting processes triggered by 34°C exposure and still evident at the end of the thermal fluctuation (38d), even when exposed to an optimum temperature value (29°C). In the constant temperature, the dominant plasmatic biochemical indicator were total proteins (PRO), reflecting an adequate nutritional and physiological status that can be inferred considering the dependence of many crustaceans on PRO as an energetic substrate (Rosas et al., 2001a; Rosas et al., 2001b). Previous studies in several shrimp species found that PRO concentrations in hemolymph function as organic reserves, serving as the basis of glycogen synthesis, the main glucose reserve (Rosas et al., 2002).

In wetlands and coastal lagoons along the northern portion of the Yucatan Peninsula, DO can temporarily reach <2 mgO_2_ L^−1^, in combination with elevated temperatures around 35°C (Herrera-Silveira et al., 2004). In adjacent marine coastal waters, DO are above 4.5 mgO_2_ L^−1^ and temperature oscillates between 24°C and 28°C at 4 m deep (Enriquez et al., 2013; Okolodkov et al., 2014). For that reason, the regulation of major fuel molecules concentration in hemolymph and metabolic pathways might reflect the ability of blue crab in tropical conditions to maintain energy expenditure across environmental gradients when they alternate between coastal waters and their adjacent ecosystems for reproduction purposes (Epifanio, 2019).

The ability of blue crab to tolerate environmental gradients may be further supported by the results obtained for total hemocyte count (THC). In this study, THC did not change significantly between DO at high temperature or across temperatures for the same DO in the thermal fluctuation. These results are different from those of Xie et al. (2021) who found that diel-cycling hypoxia can both lead to the THC reduction in the oyster *Crassostrea hongkongensis* and reported it as evidence that hypoxia may impair health of this species.

### 4.2 Stress indicators

Overall, the antioxidant defense system (ADS) was active in muscles and gills of the blue crab exposed to both (fluctuating and constant) thermal regimes to contend oxidative (OX) damage in lipids and proteins in hypoxia and normoxia. The differences detected in the patterns observed in each tissue can be explained by different functions and location within the body that make them more or less exposed to the environment (Vinagre et al., 2014).

Neither did normoxia nor hypoxia combined with high temperature significantly alter the stress response in muscle and gills compared to the constant temperature, which can be considered as the baseline scenario for each DO and tissue. This result agrees with previous findings about blue crab being well adapted to tolerate—at least temporarily—34°C, which is considered a *pejus* temperature (García-Rueda et al., 2021). The detection of some lipid peroxidation (LPO) and protein carbonylation (PO) levels in the constant temperature may seem counterintuitive with the idea of an optimum environmental regime. However, as with the concentration of free radicals in cells, low LPO and PO levels can be beneficial in cellular signaling because of their role in key processes that can improve physiological performance, like apoptosis (Wong et al., 2010; Dhabhar, 2014).

Some interesting trends were found in the OX damage indicators and the ADS enzymes in muscles of blue crab at the end of the thermal fluctuation. In animals acclimated to normoxia, LPO was neutralized when they returned to 38d (29°C), but PO was still detected at this final sampling time. PO is considered a not chemically-reversible (Dalle-Donne et al., 2006) reactive oxygen species (ROS) effect; thus, it could have accumulated through time (in organisms that returned to 29°C it doubled the value measured when they were exposed to 34°C). However, the lack of significant differences in the stress response between organisms at the end of the thermal fluctuation and those maintained at constant 29°C reflects, once again, the physiological plasticity of blue crab for environmental temperature. In this scenario, hypoxia was associated to an increase in ADS activity compared to normoxia; crabs acclimated to hypoxia presented higher levels of superoxide dismutase (SOD), the most influential variable in PCO1. This result agrees with previous findings that demonstrated how crustaceans generally maintain a high basal level of the ADS enzymes during hypoxia (Hermes-Lima and Zenteno-Savín, 2002; Welker et al., 2013). Actually, this fact is supported by the hypothesis that the increase in levels and activities of the antioxidant components during periods of hypoxia occurs in “preparation for oxidative stress” to prevent increased ROS levels during reoxygenation (Hermes-Lima et al., 2015).

In the gills of blue crab acclimated to normoxia a delayed effect of thermal stress (34°C at 24d) on OX damage associated with ROS production was observed. As in muscles, after experiencing thermal stress and decreasing temperature back to 29°C at 38d in the thermal fluctuation, the ADS was also able to neutralize LPO, but it failed to contend PO. However, the gills, significant differences were found between the stress response in blue crab that returned to 29°C in the thermal fluctuation and the rest of sampling times, including the constant temperature. The generation of ROS is presumed to be high in the gill in response to oxygen flux and diffusion because of respiration, acid-base balance, and osmotic and ionic regulation that occur in this organ (Paital and Chainy, 2010). Moreover, the gill provides the first line of antioxidant defense due to direct exposure to environmental stressors (Maciel et al., 2004), which supports a pronounced effect of thermal stress that should be contended by the ADS to maintain the functionality of this key organ. Under hypoxia, the effect of temperature was mitigated because of an increase in ADS, as previously described for muscles.

Whilst hypoxia is commonly associated with a burst in ROS (Welker et al., 2013) the increase would occur during the first hours of exposure to hypoxia and a prolonged exposure time may allow the remaining O_2_ to continue being partially reduced by complex II and III (Hermes-Lima et al., 2015). This situation could be the case of blue crab*,* considering that organisms were subjected to moderate hypoxia for weeks, which has been observed in other decapod species like the crab *Neohelice granulate*. After 10 h of hypoxia, LPO was successfully controlled thanks to the activity of the antioxidant defense system present in tissue (Geihs et al., 2014).

Carboxylesterase (CbE) and acetylcholinesterase (AChE) concentrations in muscle and gills provide more insight about the effects of fluctuating temperature between optimum and *pejus* thermal conditions for blue crab in tropical environments. CbEs are responsible for hydrolysis of a broad range of endogenous esters and play a significant role during molting (Homola and Chang, 1997). AChE is measured as a neurotoxicity biomarker (Antó et al., 2009). In muscle, when blue crab were exposed to 34°C under normoxia, thermal stress provoked an increment in AChE compared to the constant temperature, followed by a drastic decrease when blue crab returned to 29°C. Conversely, CbE levels were maintained relatively stable. Since AChE is involved in the synapse between nerve cells and muscle cells and it appears to be likely related to animals’ locomotor activity (Anita et al., 2017), higher levels at 34°C suggest an increase in neurotransmission in muscle during the escape response, which was reduced after going back to an optimum temperature of 29°C. Moderate hypoxia seemed to modulate this thermal effect. This result supports the findings for the ADS and OX damage as chronic exposure to hypoxia mitigated the effects of temperature change in muscle tissue. In the gills of blue crab acclimated to normoxia and hypoxia, differences among sampling times were mainly due to CbE, which was lower at high temperature indicating that membrane lipids in the gill could be altered by thermal stress. In contrast, AChE was maintained relatively stable among sampling times at both DO, suggesting that the nervous system linked with the gill function was maintained independently of the thermal and DO regime. However, it is worth mentioning that more detailed studies should be done in attempt to broaden the knowledge about the relationship between those enzymes, temperature, DO, and metabolism in blue crab.

## 5 Conclusion

Overall, it has been shown that temperatures that promote high values of aerobic scope (i.e., thermal metabolic scope) are associated with optimal thermal environments. Within the optimum, it has been postulated that reductions in DO decrease the energy available to meet the energy demands of routine metabolism, impairing the performance of ectotherms. In this conceptual framework, hypoxia reduces the thermal window as a consequence of the limitations posed by low DO on the cardiorespiratory system’s capacity to sustain the elevated cost of maintenance, threatening homeostasis, especially outside the optimal range (Pörtner, 2010; Pórtner et al., 2017; Sokolova et al., 2012). Contrary to this proposal, the results obtained in the present study show that hypoxia, far from reducing the performance of crabs, has a beneficial effect, motivating adaptive responses which allow a reduction in the production of free radicals, preparation of the ADS for possible re-oxygenation, and stability of the nervous system. To improve the OCLTT hypothesis and its relationship with hypoxia, it might be necessary to consider that a decrease in the metabolic rate associated with oxygen reduction does not necessarily provoke adverse effects on the life of ectotherm species like *C. sapidus* that possess the physiological mechanisms necessary to perform adequately in conditions that could be perceived as limiting.

Given the results, we conclude that in blue crab in tropical conditions, a cross-tolerance exists between temperature and DO. Moderate hypoxia attenuated the effects of high temperature on the RMR and metabolic pathways. Moreover, in blue crab exposed to temperature changes through the thermal fluctuation, when they return to an optimum temperature after experiencing thermal stress in moderate hypoxia, the degree of OX damage in muscles and gills is reduced. This general pattern attributed to DO is associated with the reduction in oxygen delivery provoked by low oxygen availability. In such circumstances, OX stress is reduced due to the role of the ADS and the decrease in ROS production associated to prolonged exposure to hypoxia.

The effect of DO on thermal tolerance of aquatic ectotherms in the literature is currently mixed (Brijs et al., 2015; Verberk et al., 2016; Jung et al., 2020).We hypothesize that in blue crab from the Yucatan Peninsula, the reduction of oxygen delivery, the modulation of energetic pathways derived from lower energy demands, and the increase in ADS activity under moderate and chronic hypoxia act as mechanisms to cope with temperature increases. This reflects the local acclimatization of blue crab to the environmental conditions that characterizes the ecological niche in the tropics. Moreover, these findings provide insights on how blue crab contend the constraints posed by abiotic variables that might be their physiological filters in aquatic environments as an invasive species.

## Data Availability

The original contributions presented in the study are included in the Supplementary Material, further inquiries can be directed to the corresponding author.
