# Supplementary figures and images for: Moderate hypoxia mitigates the physiological effects of high temperature on the tropical blue crab Callinectes sapidus
Source: Front Physiol. 2023 Jan 5;13:1089164. doi: 10.3389/fphys.2022.1089164 (PMC9849389; doi:10.3389/fphys.2022.1089164)

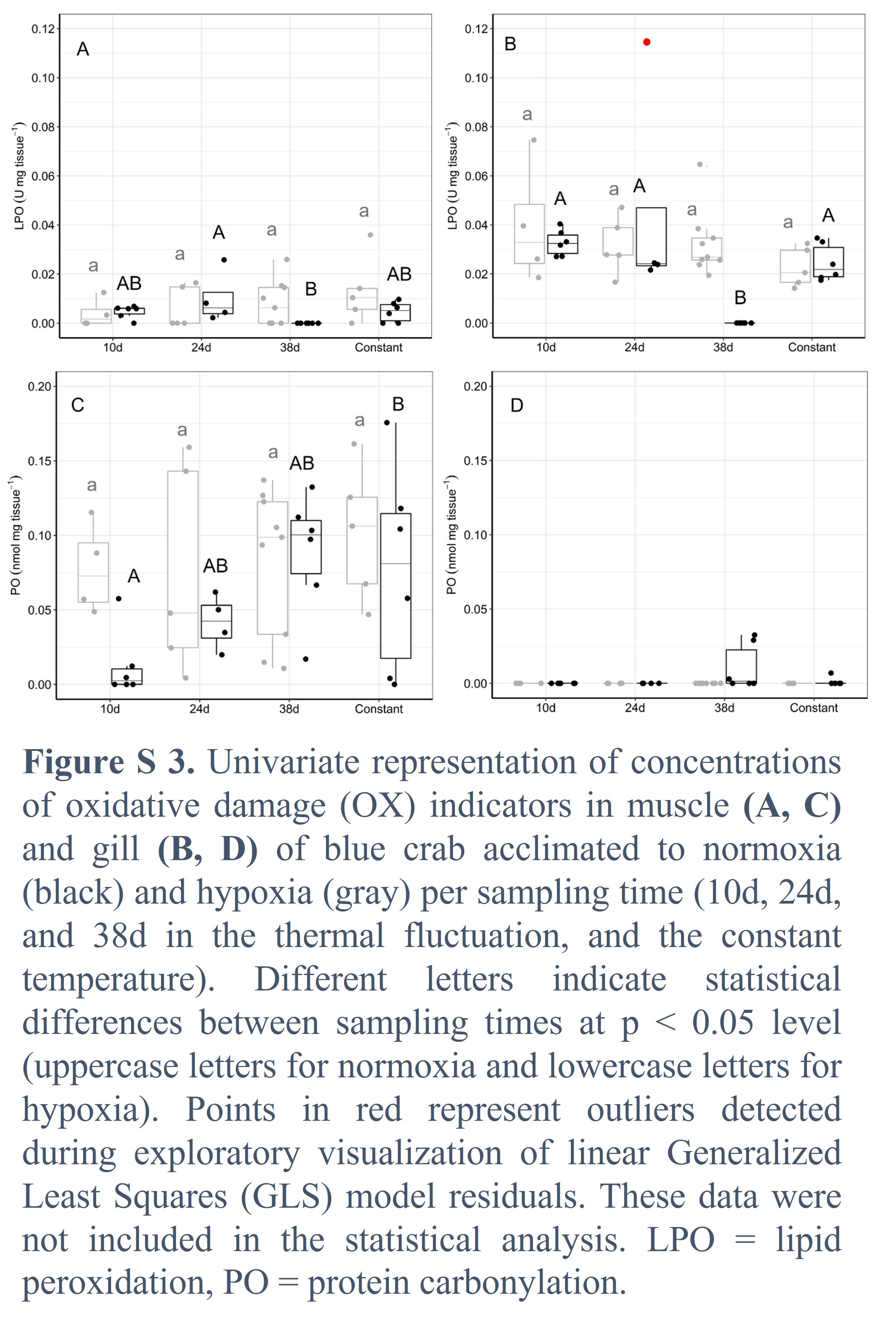

Supplement: Supplementary file 1 [file Image3.jpg]

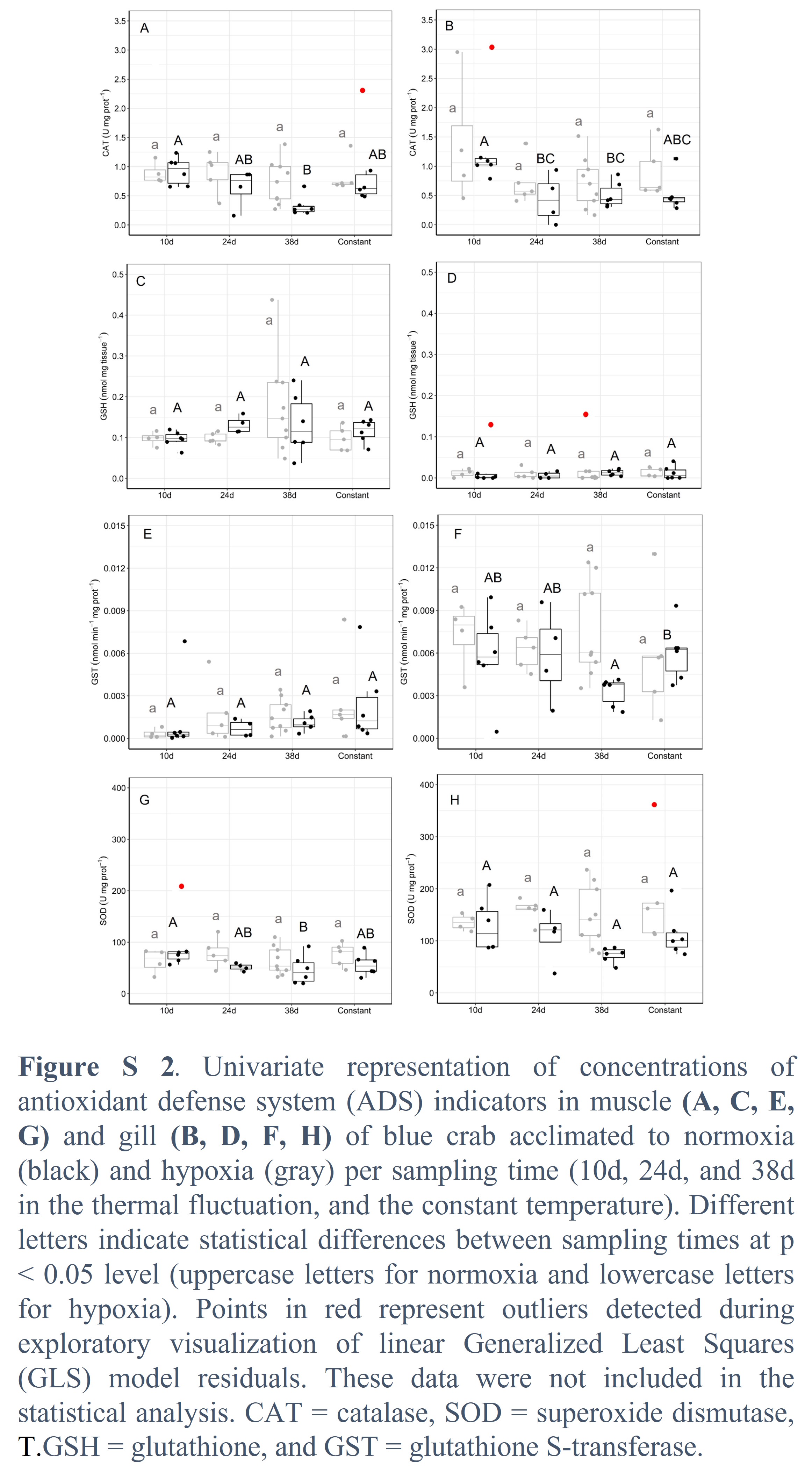

Supplement: Supplementary file 2 [file Image2.jpg]

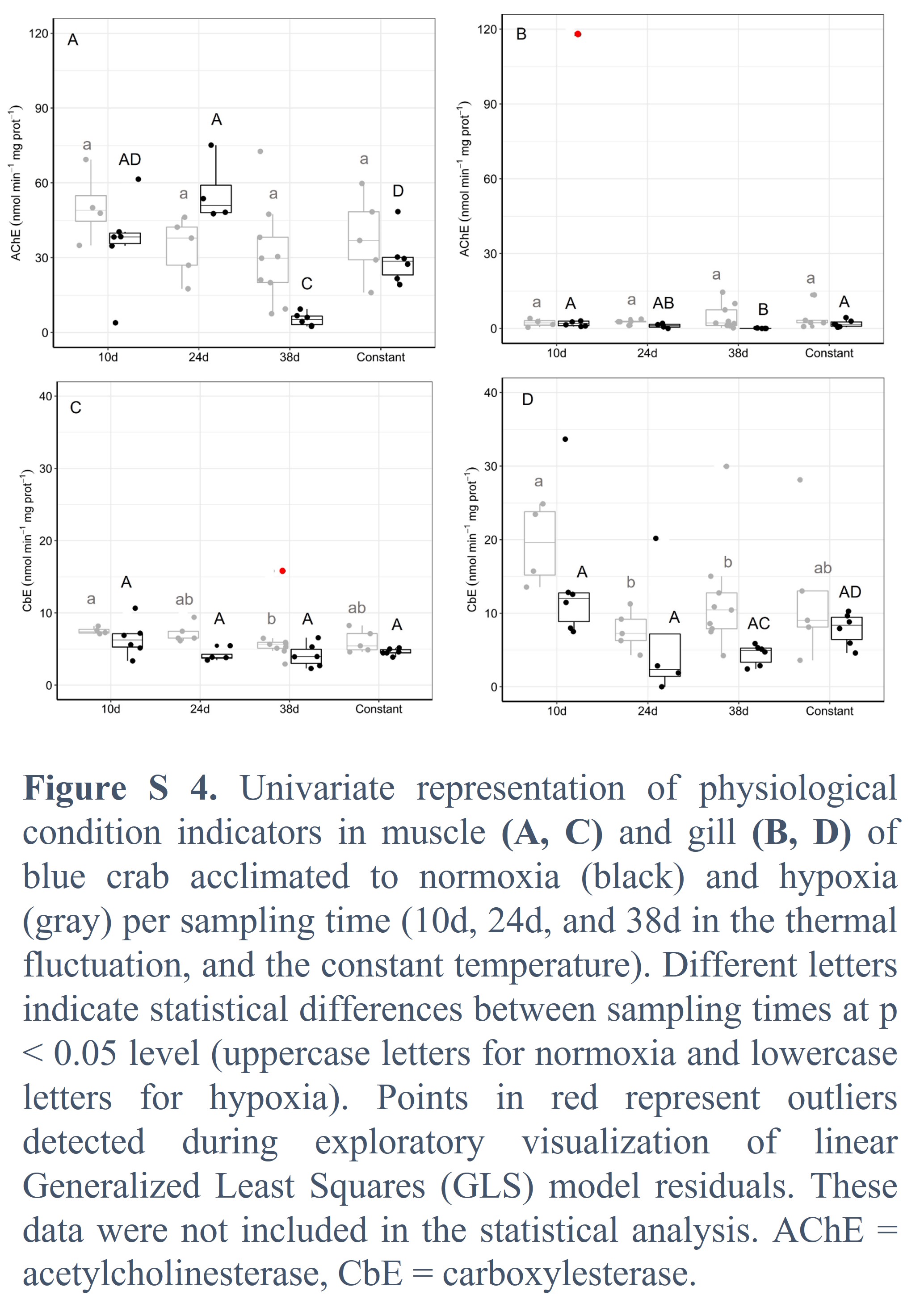

Supplement: Supplementary file 5 [file Image4.jpg]

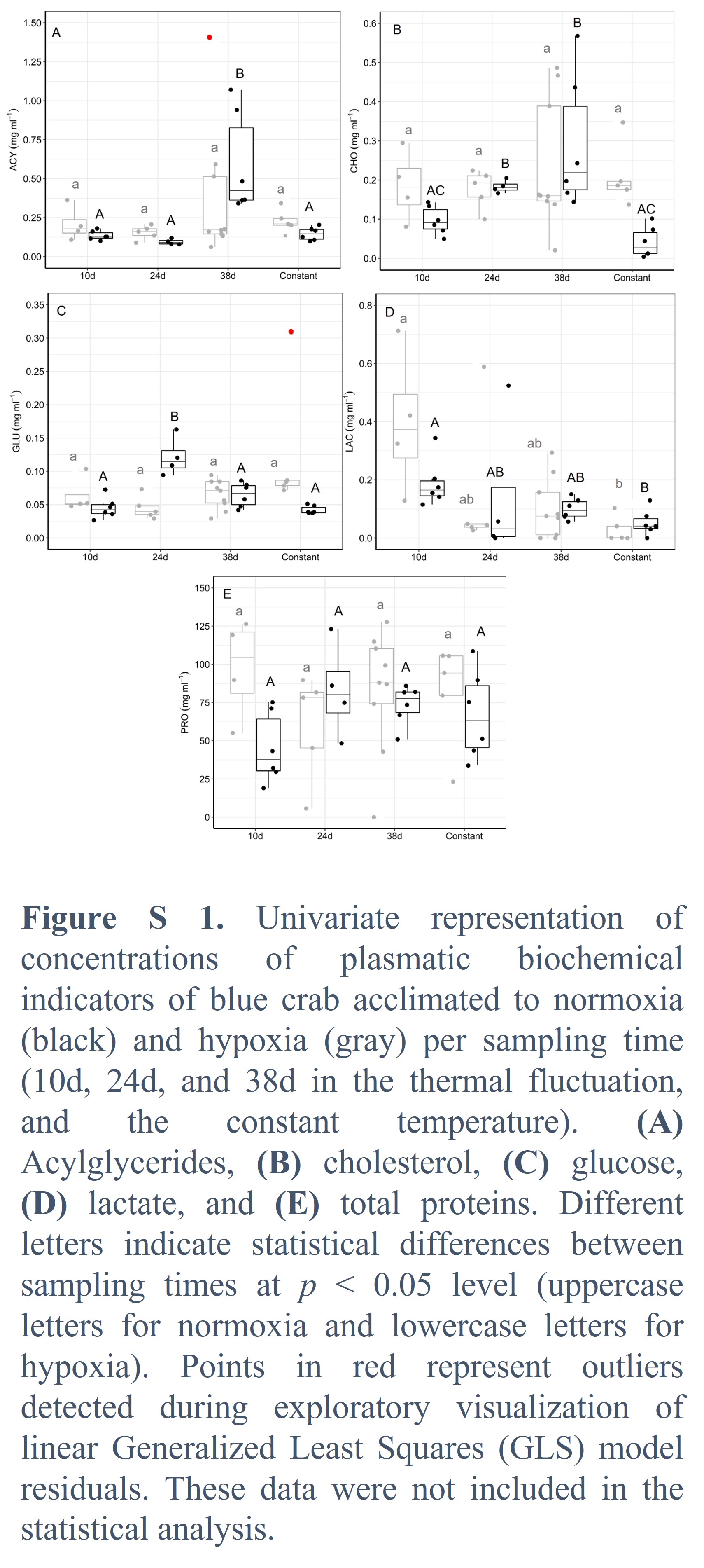

Supplement: Supplementary file 6 [file Image1.jpg]
