# Supplementary material for: Moderate hypoxia mitigates the physiological effects of high temperature on the tropical blue crab Callinectes sapidus
Source: Front Physiol. 2023 Jan 5;13:1089164. doi: 10.3389/fphys.2022.1089164 (PMC9849389; doi:10.3389/fphys.2022.1089164)
Supplement: Supplementary file 4 [file Table3.DOCX]

Table S 1. Total hemocyte count (THC) in Callinectes sapidus subjected to different thermal regimes and DO. Means with different superscripts are significantly different from each other.

| Sampling time | DO | THC (cells ml^-1^) |
| --- | --- | --- |
| 10 d | Normoxia | 1.3x10^7^ ± 4.5x10^6 a^ |
|  | Hypoxia | 4.8x10^6^ ± 6.1x10^6 b^ |
| 24 d | Normoxia | 1.1x10^7^ ± 1.6x10^6 ab^ |
|  | Hypoxia | 9.7x10^6^ ± 3.4x10^6 ab^ |
| 38 d | Normoxia | 1.2x10^7^ ± 4.7 x10^6 a^ |
|  | Hypoxia | 9.7x10^6^ ± 6.4x10^6 ab^ |
| Constant | Normoxia | 1.5x10^7^ ± 3.54x10^6 a^ |
|  | Hypoxia | 1.4x10^7^ ± 4.3x10^6 a^ |
